# Outcome of experimental stroke in C57Bl/6 and Sv/129 mice assessed by multimodal ultra-high field MRI

**DOI:** 10.1186/2040-7378-2-6

**Published:** 2010-03-15

**Authors:** Mirko Pham, Xavier Helluy, Stefan Braeuninger, Peter Jakob, Guido Stoll, Christoph Kleinschnitz, Martin Bendszus

**Affiliations:** 1 Department of Neuroradiology, University of Heidelberg, Im Neuenheimer Feld 400, D-69120 Heidelberg, Germany; 2 Department of Neuroradiology, University of Wuerzburg, Josef-Schneider-Str 11, D-97080 Wuerzburg, Germany; 3 Department of Experimental Physics 5 (Biophysics), University of Wuerzburg, Am Hubland, D-97074 Wuerzburg, Germany; 4 Department of Neurology, University of Wuerzburg, Josef-Schneider-Str 11, D-97080 Wuerzburg, Germany

## Abstract

Transgenic mice bred on C57Bl/6 or Sv/129 genetic background are frequently used in stroke research. It is well established that variations in cerebrovascular anatomy and hemodynamics can influence stroke outcome in different inbred mouse lines. We compared stroke development in C57Bl/6 and Sv/129 mice in the widely used model of transient middle cerebral artery occlusion (tMCAO) by multimodal ultra-high field magnetic resonance imaging (MRI).

C57Bl/6 and Sv/129 mice underwent 60 min of tMCAO and were analyzed by MRI 2 h and 24 h afterwards. Structural and functional images were registered to a standard anatomical template. Probability maps of infarction were rendered by automated segmentation from quantitative T2-relaxometric images. Whole-brain segmentation of infarction was accomplished manually on high-resolution T2-weighted (T2-w) RARE images. Cerebral perfusion (cerebral blood flow, CBF) was measured quantitatively by modified continuous arterial-spin-labeling (CASL) and apparent diffusion coefficients (ADC) by spin-echo diffusion-weighted imaging (DWI).

Probabilities of cortical (95.1% ± 3.1 vs. 92.1% ± 2.5; p > 0.05) and subcortical (100% vs. 100%; p > 0.05) infarctions at 24 h were similar in both groups as was the whole-brain volumetric extent of cerebral infarction. In addition, CBF and ADC values did not differ between C57Bl/6 and Sv/129 mice at any time point or region of interest.

The C57Bl/6 and Sv/129 genetic background is no major confounding factor of infarct size and cerebral perfusion in the tMCAO model.

## Introduction

The implementation of transgenic mice has also revolutionized the field of experimental stroke research in that the effects of distinct genes on stroke outcome can be easily assessed. Most of transgenic mice originate from C57Bl/6 or Sv/129 inbred strains. Moreover, these strains are also commonly used as "wild-type controls". However, there are considerable strain-related differences in the susceptibility to cerebral ischemia. Variations in cerebrovascular anatomy and hemodynamics as well as sensitivity to excitotoxicity have been identified as underlying reasons [1-7]. This may become of particular relevance when transgenic mice with mixed genetic background are compared to purebred controls. C57Bl/6 mice have been shown to be more susceptible to ischemic injury compared to Sv/129 mice in models of global (forebrain) ischemia [8] and to develop larger brain infarctions in permanent middle cerebral artery occlusion (pMCAO) [2,9]. Whether these findings also extend to the most frequently used murine stroke model, i.e. transient middle cerebral artery occlusion (tMCAO) with an intraluminal thread, is not known. Furthermore, important functional parameters of acute ischemic brain damage such as cerebral perfusion or cytotoxic edema formation have not been systematically compared between these two popular strains until now.

We recently introduced multimodal magnetic resonance imaging (MRI) at high-field strength of 17.6 Tesla as a powerful tool to non-invasively analyze the early phase of cerebral ischemia and the evolution of infarctions in individual mice over time [10]. In the present study, C57Bl/6 and Sv/129 mice were subjected to 60 min tMCAO. MRI outcome measures were chosen to characterize cerebral perfusion by continuous arterial spin labeling (CASL), hypoxic diffusion restriction by diffusion-weighted imaging (DWI), and irreversible infarction by lesion extent on T2-w and quantitative T2 relaxometric images.

## Methods

### Experimental design and animal stroke model

All procedures and animal studies were approved by the appropriate local authorities (Regierung von Unterfranken, Wuerzburg, Germany) and conducted in accordance with recommendations for the performance of basic experimental stroke studies [11]. The experimental groups consisted of 8 6-8-weeks-old male C57Bl/6 mice (Charles River, Sulzfeld, Germany) and 8 6-8-weeks-old male Sv/129 mice (Harlan-Winkelmann, Borchen, Germany) weighing 20-25 g. Animals of each group underwent tMCAO with 60 min occlusion time.

tMCAO was performed as described in detail previously [12-14]. Briefly, a standardized suture coated with silicon rubber (6021PK10; Doccol Corporation, Redlands, CA, USA) was introduced into the right common carotid artery and advanced via the internal carotid artery to the origin of the middle cerebral artery (MCA). The suture was fixed and left in situ and animals were allowed to recover. Operation time per animal did not exceed 15 min. After 60 min, animals were re-anesthetized and the suture was withdrawn to allow tissue reperfusion (tMCAO). The operations were performed under inhalation anesthesia (2.0% isoflurane in a 70%/30% N_2_O/O_2 _mixture) and the body temperature was maintained at 37°C using a servo-controlled heating pad. All animals were subsequently followed in-vivo by serial multimodal ultra-high field MRI at 2 h and 24 h.

### Multimodal ultra-high field MRI of experimental cerebral ischemia in-vivo

The detailed description of the imaging protocol can be found elsewhere [10]. A short summary of relevant parameters of the employed pulse sequences is given here. Cerebral perfusion was measured using a modified arterial spin labeling (CASL) method [15-17]. To benefit especially from increased longitudinal magnetization and the elevation of the T1 relaxation time for detailed anatomical mapping of CBF and group analysis, all measurements were performed at ultra-high field strength (17.6 T, 750 Hz, Biospin, Bruker BioSpin GmbH, Ettlingen, Germany). Image maps of cerebral perfusion were calculated on a pixel-by-pixel basis according to Detre et al. [15]. The degree of the inversion efficiency was assumed to be alpha = 0.7 [18,19], and the brain-blood partition coefficient value for water lambda = 0.95 mL/g [20]. Parameters for the fast spin-echo imaging sequence (RARE) were: echo train length (ETL) = 8, effective echo time TE_eff _= 17.2 ms, repetition time TR = 1 s, slice thickness 1.5 cm, FOV 2.5 × 2.5 cm, matrix of 64 × 64 voxels. The signal was averaged over 12 repetitions resulting in a total acquisition time of 9.5 min.

DWI was performed with a pulsed-field gradient Setjskal-Tanner-like multislice spin echo sequence with diffusion sensitization along the slice direction [21]. Images with different b-values, 0 and 800 s/mm^2^, were acquired to allow for the calculation of apparent diffusion coefficient (ADC) maps of brain water. Whole brain coverage was achieved by thirteen coronal slices acquired with a matrix size of 64 × 64, FOV 2.5 × 2.5 cm, in plane resolution 282 × 282 μm, slice thickness = 0.5 mm, interslice distance = 1 mm, TE/TR = 22.3/2000 ms. Repeated measurement of the b = 800 s/mm^2 ^DWI experiments (number of repetition NR = 3) resulted at an overall acquisition time for diffusion weighted experiments of 8 min. ADC maps were calculated by applying the common equation ADC = -0.00125 × ln (SI_B800_/SI_B0_).

T2 relaxometric mapping was performed for the in-vivo delineation of infarcted brain tissue at 24 h. Single slice T2-w imaging was performed using a Carr-Purcell-Meiboom-Gill (CPMG) multi-spin echo sequence collecting 32 echoes at TR/TE = 4.2/2000 ms. T2 relaxation times constants were calculated voxel-wise by fitting the intensities of the 20 first echoes to a monoexponential model. In addition, a strongly T2 weighted 2D turbo spin-echo sequence was acquired for high resolution anatomical imaging (RARE factor 16, TR = 8 s, effective TE = 56.44 ms, 2 averages, 13 coronal slices with an image matrix of 128 × 128 were acquired, FOV = 2.5 cm × 2.5 cm, slice thickness = 0.5 mm, interslice distance = 1 mm, overall acquisition time of 1 min). The center position of the 1.5 mm slab for measurement of CBF, T1 and T2 relaxometric maps each with the exact same geometry was centered at the bregma as the operational definition of the central MCA territory.

At the host console measurements and data processing were performed with the ParaVision software (version 3.02, Bruker BioSpin GmbH, Ettlingen, Germany). Further image calculation and fitting procedures were done using *MATLAB*^® ^(The Mathworks Inc., Natick, MA, USA).

During MRI measurements, mice were anesthesized by 2% isoflurane in medical air (21%). The respiratory rate was monitored using an air-balloon positioned ventrally underneath the mouse body. The body temperature was constantly measured on the body surface and actively maintained at 37°C.

### Statistical and image analysis

The extraction of brain tissue from the scalp and skull was done by manual segmentation for each subject and time point. Packages from the FMRIB Software Library FSL (version 4.1) [22] were used for motion correction, registration (FLIRT) [23], and statistical image analysis. Intra-subject linear alignment and registration to a common standard template [24] was achieved by a step-wise affine procedure with six degrees of freedom.

**Figure 1:**
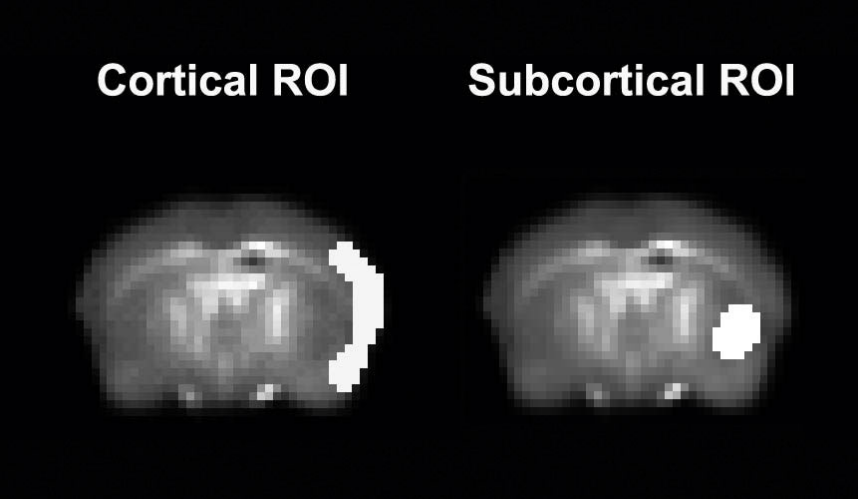
**Region-of-interest masks**. On the left the central cortical territory of the middle cerebral artery (MCA) is delineated by the white overlay region according to the corresponding coordinates in atlas space, and the deep subcortical territory of the MCA is displayed on the right.

For quantitative group comparisons, selected regions of interest (ROIs) were delineated in atlas space: 1) the cerebral cortex in the center of the MCA territory and 2) the subcortex including the ipsilateral caudoputamen and pyramidal tract (Figure 1). Statistical analysis of ROIs was done by non-parametric pairwise comparisons using the Wilcoxon matched-pairs signed-rank test. The risk of cerebral infarction was determined on within-group probability maps calculated by averaging the binary segmentations of healthy versus infarcted brain tissue within-subject. Automated binary segmentation of cerebral infarction was performed by applying a threshold of 34 ms T2 relaxation time on the T2 relaxometric maps at 24 h as demonstrated previously [10]. Manual input was given only for the removal of intraventricular cerebrospinal fluid. Finally, whole-brain volumetric analysis of infarction was done by manual segmentation on the images of the T2-w RARE sequence. Values are always given as mean ± standard error of the mean (SEM).

## Results

### Manual whole-brain segmentation of infarction

**Figure 2:**
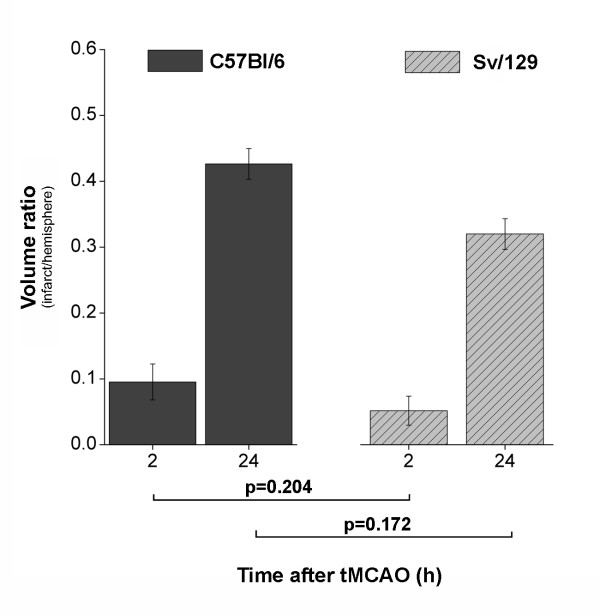
**Group means of infarct volume ratios (infarction/hemisphere)**. Pairwise comparisons between 8 C57Bl/6 and Sv/129 mice did not reveal any statistical differences for the 2 h (p = 0.204) or 24 h time point (p = 0.172) after tMCAO. Error bars denote standard errors of the mean.

Volumetric extents of cerebral infarction as delineated manually on T2-w RARE imaging are given as volume ratios (infarction/hemisphere) and were as follows for the C57Bl/6 mice: at 2 h 0.1 ± 0.03 and at 24 h 0.43 ± 0.02. For the Sv/129 mice, values were 0.06 ± 0.03 at 2 h and 0.37 ± 0.03 at 24 h. No statistical differences were found when comparing pairwise-matched groups (Figure 2).

### Probability maps of infarction by quantitative T2 thresholds

**Figure 3:**
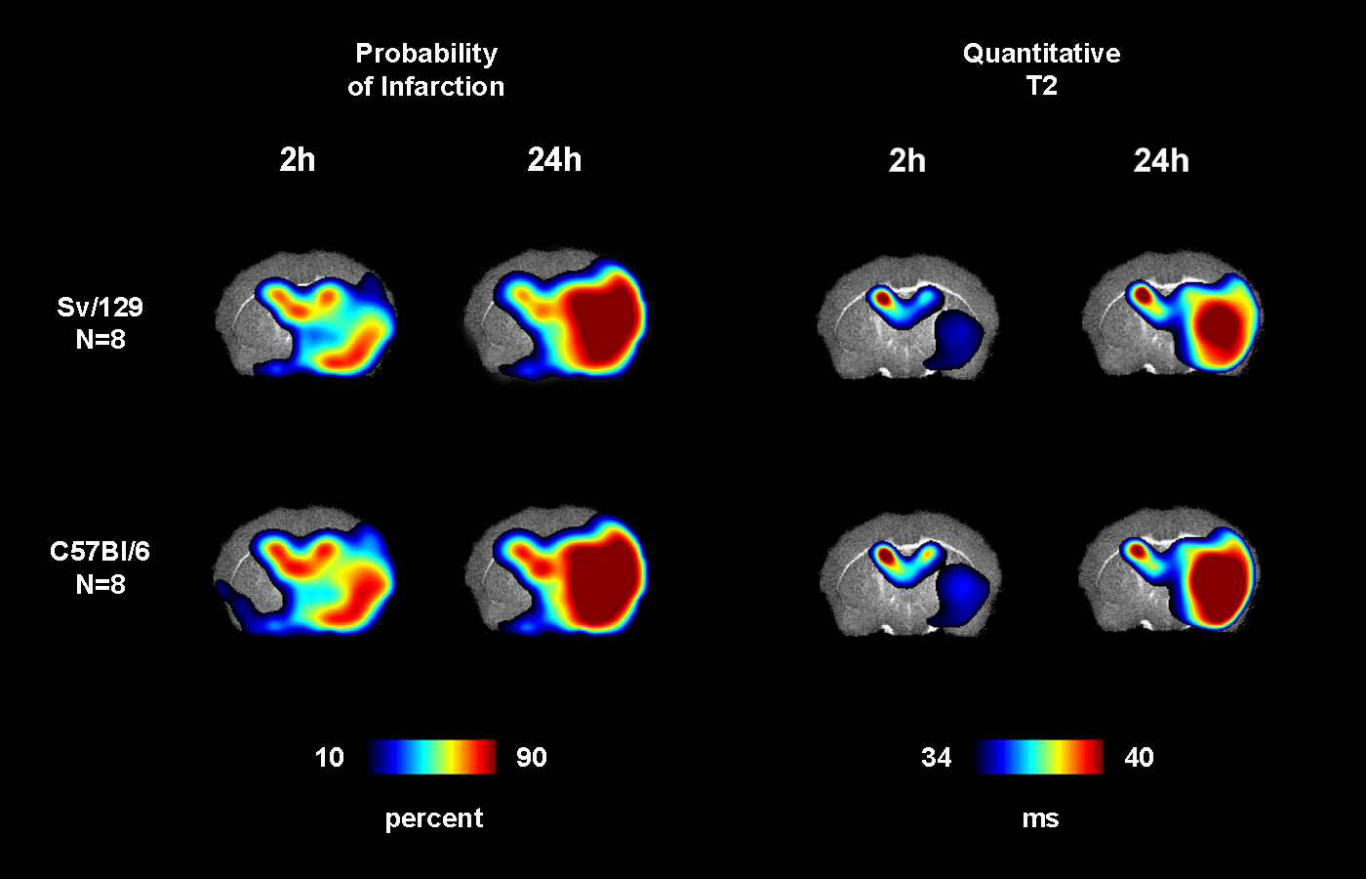
**Color coded group means of probability of infarction and quantitative T2 values**. For both groups probability of cortical and subcortical infarction was similar. Note that subcortical infarction is evident on T2 maps already 2 h after tMCAO. The segmentation threshold for infarction was set to 34 ms.

Automated segmentation of infarction for each individual animal was performed with a threshold of a quantitative T2 value of 34 ms as described previously [10]. The probabilities of infarction in atlas space are given in Table 1 for both groups, time points and corresponding subcortical and cortical ROIs. There was no significant difference in the probability of infarction between both groups at 2 h (p = 0.4) or 24 h (p = 0.93). The spatial distribution of the different probabilities of infarction is demonstrated on color-coded probability maps and relaxometric T2 maps (Figure 3). Interestingly, subcortical infarctions (basal ganglia) were present already 2 h after stroke onset corresponding to the insufficient collateral blood supply in these areas. After 24 h infarction has extended to the whole MCA territory including the cortex.

**Table 1:** Group means of infarct probabilities (%).

	129/Sv		C57BL/6	
	**2 h**	**24 h**	**2 h**	**24 h**

Cortex	53.3 ± 10.1	92.1 ± 2.5	60.7 ± 10.4	95.1 ± 3.1
Subcortex	67.8 ± 12.8	100	79.1 ± 11.3	100

Values are given for both cortical and subcortical ROIs and both time points after tMCAo. Results of statistical pairwise comparisons are given in the text and did not significantly differ between groups for any time point. Standard errors of the mean are denoted by ±.

### Analysis of functional ischemic outcome parameters

Pairwise comparisons between group means of quantitative CBF values were not significant for any time point or region of interest. Two-sided p-values for matched pairwise comparisons between C57BL/6 and Sv/129 mice were: subcortical ROI at 2 h p = 0.7 and p = 0.53 at 24 h; cortical ROI at 2 h p = 0.64 and p = 0.86 at 24 h.

**Figure 4:**
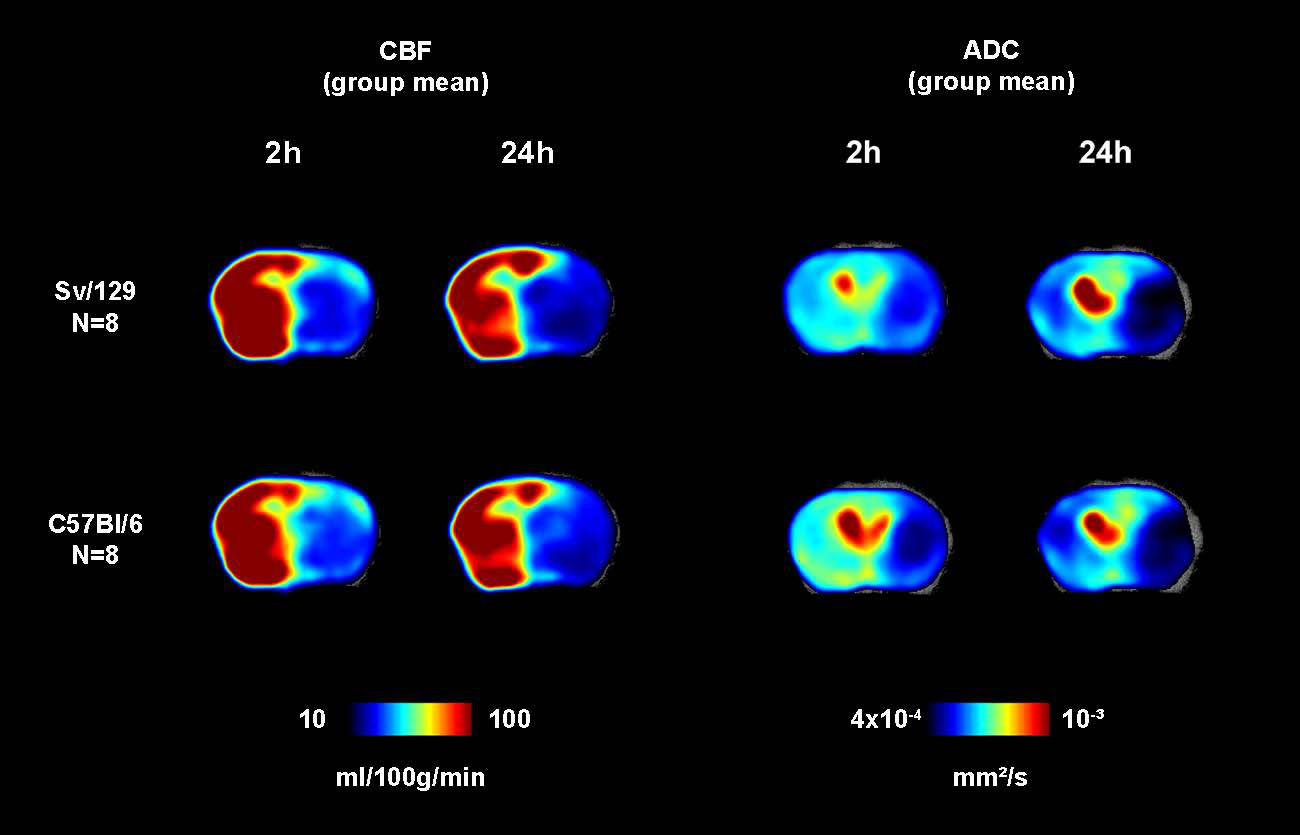
**Color maps of group means for CBF and ADC**. Both are functional parameters cerebral ischemia indicating cerebral perfusion and cytotoxic diffusion restriction. Severe cortical and subcortical hypoperfusion (CBF maps) over time was observed in both groups. ADC maps show the transition from imminent to definite cerebral infarction involving cortical and subcortical territories. No significant differences between the strains were observed. Error bars denote standard errors of the mean.

Similarly, ADC values in the same ROIs showed no statistical differences between group means. Two-sided p-values for matched pairwise comparisons between C57BL/6 and Sv/129 mice were: subcortical ROI at 2 h p = 0.34 and at 24 h p = 0.91; cortical ROI at 2 h p = 0.28 and at 24 h p = 0.63. Quantitative mean values for CBF and ADC are given in Table 2. Figure 4 displays the spatial distribution of functional outcome measures on color maps. Again, infarctions in both groups evolved from the subcortical to the cortical areas over time.

**Table 2:** Group means of functional ischemic outcome measures: CBF and ADC.

		Sv/129	C57Bl/6
CBF (ml/100g/min) 2 h vs. 24 h	Cortex	34.1 ± 4.7 vs. 22.3 ± 2.9	38.7 ± 4.8 vs. 25.2 ± 3.7
	Subcortex	30.4 ± 4.5 vs.20.8 ± 2.7	32.8 ± 4.6 vs. 23.4 ± 3.5

ADC (mm^2^/s*10^-4^) 2 h vs. 24 h	Cortex	7.8 ± 1.0 vs. 5.8 ± 0.5	7.8 ± 1.0 vs. 5.7 ± 0.6
	Subcortex	7.3 ± 1.0 vs. 5.4 ± 0.5	7.2 ± 1.1 vs. 5.3 ± 0.4

Values are given for both cortical and subcortical ROIs and compared between both time points. P-values of statistical comparisons for CBF and ADC between groups were not significant and are given in the text. Standard errors of the mean are denoted by ±.

## Discussion

As principle finding, we here show that C57Bl/6 and Sv/129 mice, the two most frequently utilized strains in experimental stroke research, behave similar in the tMCAO model regarding critical functional and structural parameters of infarct development. Volumetric extents and probability of cerebral infarctions did not significantly differ between the two groups as assessed by multimodal ultra-high field MRI as did cerebral perfusion and diffusion restriction of free water.

Strain-related differences between C57Bl/6 and Sv/129 mice have already been investigated in models of global and permanent focal cerebral ischemia. C57Bl/6 mice challenged by transient bilateral common carotid artery occlusion in the presence or absence of systemic hypotension developed more severe global forebrain ischemia than Sv/129 mice [7,8]. Results after permanent MCAO have been inconclusive. While some reports described larger infarctions in C57Bl/6 mice in direct comparison to the Sv/129 strain [2,9], others could not confirm this observation [4]. We further extend these findings to the most frequently used model of focal cerebral ischemia, i.e. tMCAO with an intaluminal thread. Here, 60 min of focal ischemia had no differential effect on definite infarct volumes on day 1. Importantly, diffusion restriction of free water as an early marker of ischemic cell damage and cytoxic edema likewise occurred similar in both strains. This implies that the C57Bl/6 or Sv/129 genetic background is no major confounding factor of infarct evolution in the acute phase after tMCAO. Whether this also applies to later stages of infarct development or different MCA occlusion times, however, still needs to be assessed.

Besides infarct volume, regional cerebral blood flow (rCBF) is frequently chosen as readout parameter in experimental stroke studies. Both critically depend on the composition of the cerebral vasculature which is known to vary considerably between different mouse strains [1-3]. The posterior communicating artery is absent or hypoplastic in more than 90% of C57/Bl6 mice, but in less than 50% of Sv/129 mice, which is also supported by in-vivo MRI data [4,25]. Moreover, the MCA territory seems to be larger in C57/Bl6 as compared to Sv/129 mice [26]. In contrast, pial anastomoses between the anterior and middle cerebral artery territories are of similar number and diameter [26]. PWI produced comparable results in C57/Bl6 and Sv/129 mice subjected to tMCAO indicating that the reported anatomical variations in the Circle of Willis do not translate into relevant functional differences in cerebral blood supply. Interestingly, infarction of subcortical areas (e.g. basal ganglia) was present as early as 2 h after tMCAO in both groups as assessed by various structural and functional MRI parameters. Early completed infarction in this subcortical territory of the MCA corresponds to the anatomically limited collateral blood supply of this region [27]. In contrast, severe hypoperfusion was observed in the cortical grey matter of the MCA territory at the same time point (2 h). Irreversible infarction followed only at 24 h determining this region as tissue-at-risk with a prolonged capacity for survival because of a richer collateral network through pial anastomoses. These observations emphasize the need for fast therapeutic interventions in ischemic stroke.

In summary, the C57/Bl6 and Sv/129 genetic background is no major confounding factors of stroke size and cerebral perfusion in the tMCAO model. Multimodal ultra-high field MRI is a valuable tool to non-invasively assess important structural and functional parameters of brain infarction in mice and can help to increase the validity of experimental stroke studies.

## Competing interests

The authors declare that they have no competing interests.

## Authors' contributions

MP, CK and SB wrote the paper. CK induced cerebral infarctions; MP and XH performed MRI measurements. MB, GS and PJ designed the study and revised the paper. All authors read and approved the final manuscript.
